# Efficacy and Clinical Outcomes of Minimally Invasive Direct Thoracic Interbody Fusion: A Retrospective Analysis

**DOI:** 10.7759/cureus.35681

**Published:** 2023-03-02

**Authors:** Hamid R Abbasi, Nick Storlie, Josh Gonzalez, Mitch A Rusten, Ziyang Ye, Nicholas Van Halm-Lutterodt, Michael Jaeger

**Affiliations:** 1 Spine Surgery, Inspired Spine Health, Burnsville, USA; 2 Neurological Surgery, Tristate Brain and Spine Institute, Alexandria, USA; 3 Research, Inspired Spine Health, Burnsville, USA; 4 Research, Tristate Brain and Spine Institute, Alexandria, USA; 5 Neurosurgery, Beijing Tiantan Hospital, Capital Medical University, Beijing, CHN; 6 Orthopaedics, Beijing Chaoyang Hospital, Capital Medical University, Beijing, CHN

**Keywords:** disc disease, level 3 retrospective cohort study, interbody fusion, minimally invasive surgery, operative surgical procedures, spine surgery, thoracic spine, spinal fusion

## Abstract

Introduction: A unique surgical approach - the minimally invasive direct interbody fusion (MIS-DTIF) - was previously introduced in our proof-of-concept study, which included four patients who underwent thoracic interbody fusion below the scapula at the T6/7 vertebral level. However, due to the novelty of this method, a report of associated operative parameters such as pain, function, and clinical outcomes from an expanded patient cohort was needed to assess the validity of our results.

Materials and Methods: Following IRB approval, data were analyzed retrospectively from electronic health records between 2014 and 2021. Inclusion criteria were patients ≥18 years old who underwent minimally invasive thoracic interbody fusion using the MIS-DTIF technique for at least one vertebral level. The primary outcomes included demographic/radiographic features (e.g., age). Secondary outcomes included perioperative clinical features (e.g., preoperative and ≥1-year final follow-up (FFU)). Tertiary outcomes included perioperative complications. Both preoperative and FFU patient-reported pain and functional outcomes (ODI scores) were analyzed using t-tests to establish significance.

Results: A total of 13 patients who underwent MIS-DTIF surgery were observed, with eight male patients and five female patients. The average age was 49.2 years, with an average BMI of 30.5 kg/m^2^. Of the surgeries included, the majority (69.23%) were 1-level thoracic vertebrae fusions - with 2-level fusions and ≥ 3-level fusions accounting for 15.38% and 15.38% of cases, respectively. The mean operative time was 58.9 ± 19.9 minutes, with an average fluoroscopy time of 285.7 ± 126.8 seconds and an average actual blood loss volume of 109.0 ± 79.0 mL. The average hospital length of stay was 1.1 (±1.7) days, and no clinically significant perioperative complications were observed in this patient cohort. The average follow-up period was 12.1 ± 9.6 months, with preoperative and FFU back pain visual analog scale (VAS) scores showing highly significant improvement (*p<0.001*). In addition to pain reduction, quality of life improvements was noted, with significant differences in some of the ODI domains between preoperative and FFU scores (*p<0.05*), as well as the overall total score between preoperative and FFU ODI assessment (*p<0.001*) - both of which reflect increased patient function and decreased disability.

Conclusion: This study provides further evidence for the safety and efficacy of the MIS-DTIF approach for surgical management of symptomatically refractory patients with thoracic disc herniation or stenosis owing to degenerative disc disease or compression fractures. Additionally, the data gathered suggests that this minimally invasive procedure offers many clinical benefits, including less tissue damage, decreased intraoperative blood loss, shortened surgery time, and shortened hospital length of stay. Finally, in addition to significant pain intensity improvement, this study showed that treated patients highly benefited from ‘sleeping’ and ‘return-to-work’ domains and other ODI functional domains in activities of daily living (ADLs). More clinical studies are recommended in larger patient cohorts to ascertain the findings reported in this study.

## Introduction

Chronic back pain (CBP) is a common debilitating condition impacting patient populations and is a leading cause of disability globally [1,2]. This condition also has large economic implications, with spine expenditures of $85.9 billion in 2005 and trending upwards [3]. Painful thoracic disc herniations are relatively rare and makeup only about 0.5% to 4.5% of spinal disc herniation or stenosis [4]. While the condition is often asymptomatic and found incidentally, thoracic discogenic syndrome can lead to axial and radicular pain, as well as myelopathic signs and symptoms.

The relatively narrow canal of the thoracic spine levels makes this section of the spine more susceptible to the development of symptomatic stenosis even from small herniation [4-6]. Central herniations usually result in myelopathy symptoms, and lateral herniations lead to radicular signs and symptoms such as mid-back pain, epigastric, and flank pain [7]. Due to inadequate assessment of symptoms and clinical presentation, as well as the rarity of occurrence, this condition is often underdiagnosed. Similar to lumbar discs, the symptoms are initially managed conservatively by a combination of NSAIDs, physical therapy, rest, epidural injections, and other non-operative treatment modalities. Given the uncommon nature of this disease, there is a significant lack of quality clinical studies to investigate patient improvement and outcomes from non-operative treatments and/or surgery [8]. Nevertheless, surgery remains the last resort reserved for symptomatic cases that are unresponsive to conservative therapy options - or for patients whose conditions deteriorate quickly and are complicated by neurological deficits [9].

Various techniques have been utilized in the surgical treatment of herniated thoracic discs, including transpedicular costotransversectomy, extracavitary, and transthoracic approaches [7,8]. There are limitations and benefits to each, but, in general, the thoracic vertebral column is difficult to access and often requires manipulation of ribs and thoracic viscera. This is usually associated with increased pain, lengthy hospital stays, and higher morbidity/mortality, which has been reported to be up to 30%. Despite these numerous technical complications, there is no proven benefit of one approach over the other, nor is there high-quality evidence to report quality improvement in symptom control using a specific approach [9-14].

Minimally invasive direct thoracic interbody fusion (MIS-DTIF) is a recently described novel approach that aims to minimize the pain and disability associated with thoracic disc herniation or stenosis while providing effective and long-lasting pain relief [14-19]. We, therefore, aimed to evaluate the perioperative clinical outcomes, as well as pain and disability outcomes in spine patients diagnosed with degenerative thoracic disc herniation or thoracic stenosis that was surgically managed by the MIS-DTIF procedure.

## Materials and methods

Study design

This study is a retrospective cohort review of 13 patients who underwent the MIS-DTIF procedure between 2014 and 2021. This procedure was performed by a single surgeon in five different health institutions in Minnesota: [(1) Douglas County Hospital; (2) Fairview Ridges; (3) North Memorial; (4) River View Health; (5a) Maple Grove Hospital; (5b) Maple Grove Surgery Center, and U.S. Institutional review board (IRB) exemption was granted by Pearl Pathways IRB. Inclusion criteria for this study were patients that were ≥18 years old who underwent minimally invasive thoracic interbody fusion using the MIS-DTIF technique for at least one level of the procedure. MIS-DTIF fusion was performed by the first author (Dr. Hamid R. Abbasi) between 1/1/2014 and 12/31/2021 on patients meeting the diagnosis of thoracic pain (i.e., clinical diagnostic criteria with characteristic history who have radiographic and clinical evidence of thoracic disc disease and herniation) after conservative therapy had failed and other alternatives had been discussed with the patient. Patients included had at least six months of chart data available, and patients with medical comorbidities such as diabetes were eligible for inclusion.

All included patients in the study underwent at least 3 to 6 months of conservative therapy before being considered candidates for surgery. Preoperative imaging included magnetic resonance imaging, X-ray of the thoracic spine, and typically a discogram and post-discogram computed tomography (CT) scan. Patients were indicated for MIS-DTIF if they were found to have radiologic and clinical evidence of thoracic disc disease and herniation with failure to respond to conservative therapy for six months.

Criteria for exclusion included: patients participating in an investigational program with interventions outside of routine clinical practice; pregnancy; the presence of the following diagnoses: osteomyelitis, bone tumors, patients with secondary gain such as work injury and worker compensation claims, and significant psychosocial issues making an objective evaluation of the patient impossible. Other exclusion criteria for MIS-DTIF included significant scoliosis and pathology above the T5/6 vertebral body level due to obstruction by the scapula. 

Our database returned a total of 34 patients who had undergone MIS-DTIF throughout the observed period. Of the initial 34 patients who underwent MIS-DTIF in our database, 14 were excluded due to the above-mentioned exclusion criteria resulting in 20 patients that had enough preoperative data. Of these 20 patients, only 13 had appropriate postoperative follow-up data to draw results from, resulting in our core study group. 

Surgical approach

**Figure 1:**
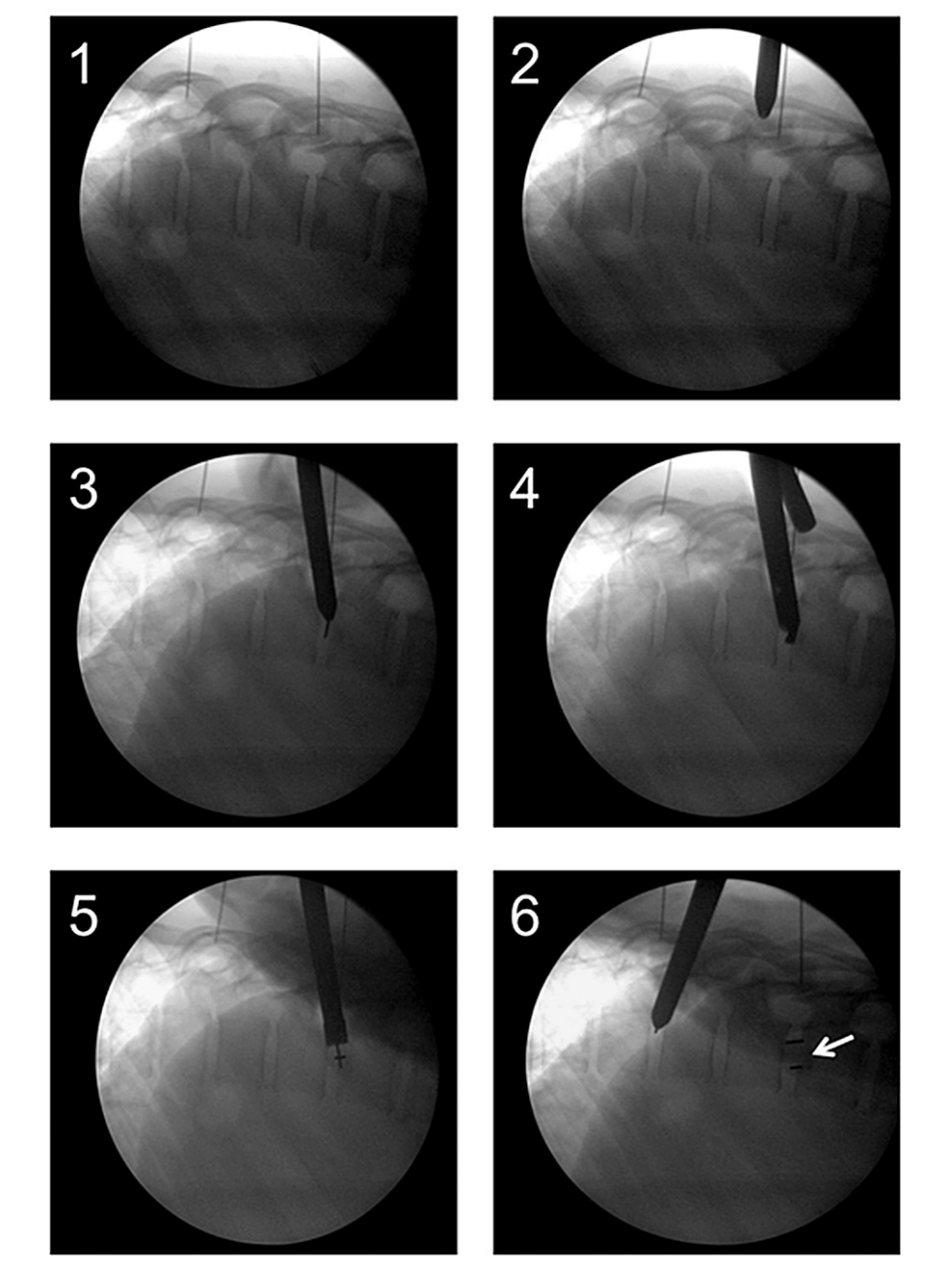
MIS-DTIF in the lateral fluoroscopic view 1) Localization, 2) Approach, 3) Disk entry, 4) Disk removal using the drill, 5) Cage entry, 6) Cage entry completed (arrow)

**Figure 2:**
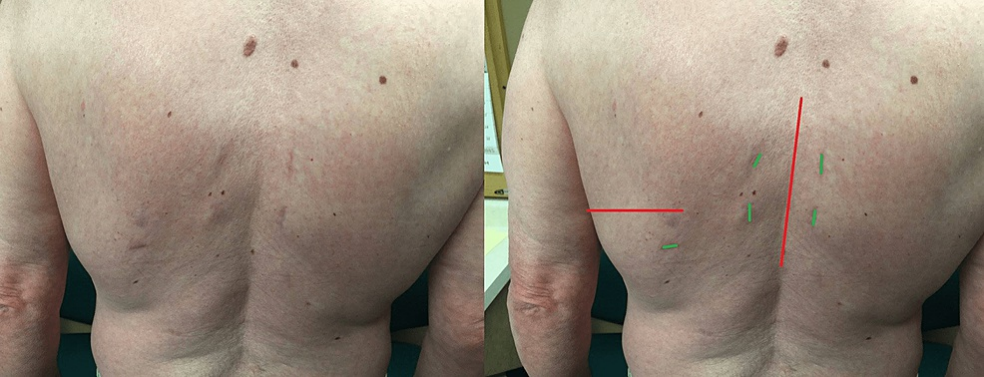
MIS-DTIF back region markings for incisions The incision point is chosen to give a 45º angle of approach to the spine.

On the posterior axillary line, a 1.5-cm surgical incision was made just above the correctly identified rib. Then, under biplanar fluoroscopic imaging, a blunt 8-mm cannulated probe was inserted into the pleural cavity and traversed along the rib with continued rib contact towards the rib head within the pleural cavity and finally placed on the capsule of the disc of interest. Figures 1, 2. Then, utilizing fluoroscopic guidance, we introduced the sharp-tip K-wire into the cannulated probe and entered it into the disc space, after which we set the working tube over the blunt cannulated probe and into the disc space. This established the working channel from the skin into the disc space. During the entire surgical procedure, a direct link from the skin to disc space, sealed by the skin and intercostal ligament and capsule of the disk, was conserved through a 10-mm working tube. The approach with a bilateral fluoroscopic view, discectomy, and cage placement, as well as posterior pedicle screw fixation, are all well described and documented in our previously published proof of concept study [14].

Outcome measures

To establish a follow-up endpoint, we evaluated patient clinical outcomes from their last preoperative clinic visit and at ≥6-month postoperative visits, defined as at least 150 days after surgery to allow for flexible patient scheduling. The primary outcomes included baseline demographic features [age, gender, body mass index (BMI), and number of thoracic levels fused]. Secondary outcomes included perioperative clinical features [preoperative and ≥6-month final follow-up (FFU) visual analog scale-[VAS] and Oswestry Disability Index-[ODI] scores; mean operation time (MoT), In-Out of operating room time, hospital length of stay (HLoS), fluoroscopy time (FT), and actual blood loss (ABL). The MoT, ABL, and FT were recorded during and immediately following surgery and stored in our electronic database systems. The ABL was calculated by measuring the weight of blood-saturated sponges and subtracting the dry weight of the sponges, resulting in actual external blood loss. Tertiary outcomes included perioperative complication outcomes. Any perioperative complication events (wound infection, neurologic complications, reoperation, hardware issues, pseudoarthrosis or nonunion, other medical or surgical-related complications) were recorded intra-operatively, immediately-postoperatively, and at subsequent follow-up visits.

Statistical analysis

Continuous data were reported as means and their respective standard deviations (X̅±SDs), while dichotomous variables were presented as percentage frequencies [n (%)]. The comparison between baseline and last patient visit outcomes was analyzed by using t-tests to establish any significance between preoperative and FFU pain intensity (VAS) and functional outcome (ODI) scores. Data analysis and visualization were performed in the ‘R’ version 3.4 statistical software package. Significance was set at (p<0.05).

## Results

Primary outcomes

Demographics

A total of 13 eligible patients who underwent MIS-TLIF surgery were observed in this study period. The average age of these patients was 49.2 years, with a female incidence of 35.5% (n=5/13). The average BMI was 30.5kg/m2. Regarding the frequency of thoracic levels fused, we observed 69.2% (n=9/13) for 1-level, 15.4% (n=2/13) for 2-level, and 15.4% (n=2/13) for ≥ 3-level fusions (Table 1).

**Table 1:** Mean perioperative outcomes of the study group

	Overall
n	34
BMI (mean (SD))	31.2 (5.8)
Age (mean (SD))	51.3 (13.9)
Blood Loss (mean (SD))	144.1 (121.8)
OR Time (mean (SD))	74.7 (38.6)
Fluoroscopy Time (mean (SD))	252.9 (117.8)
Hospital Stay (mean (SD))	1.1 (1.7)
Thoracic Levels Fused n (%)	
1	17 (50.0)
2	10 (29.4)
3	5 (14.7)
4	1 ( 2.9)
5	1 ( 2.9)

Secondary outcomes

Other Perioperative Clinical Variables

The average MoT of MIS-DTIF surgery in these patients was 58.9 ± 19.9 minutes, and the In-Out operating room time was 109.9 ± 25.9 minutes. The average FT during the procedure was 285.7 ± 126.8 seconds. The average ABL in these patients was 109.0 ± 79.0 mL, and the average HLoS was 1.1 ( ± 1.7) days. The average follow-up period was 12.1 ± 9.6 months (Table 1).

Patient-Reported Pain, Functional and Disability Outcomes

A mean back pain VAS score of 7.31 ± 2.50 cm in 13/13 patients at preoperative significantly decreased to 2.08 ± 2.30 cm in 10/13 patients at FFU (p<0.001). We observed n=13/13 ODI outcomes in the preoperative phase with a total score of 47.23 ± 20.49 points n=10/13 ODI outcomes at ≥6 months FFU with a total score of 29.64 ± 18.86 points, (p<0.001). Within the ODI domains, we also observed significant differences between preoperative and FFU categories of ‘pain intensity’, [2.77 vs. 1.40], ‘personal care’, [2.08 vs. 1.20], ‘walking’, [1.92 vs. 1.10], ‘sleeping’, [2.62 vs. 1.70], ‘social life’, [2.54 vs. 1.70], ‘traveling’, [2.23 vs. 1.24], and ‘housework/employment’, [2.15 vs. 1.60] scored points, (p<0.05), respectively (Table 2).

**Table 2:** Oswestry Disability Index (ODI) variables

	post	pre	p	
n	15	21		
Pain (mean (SD))	3.07 (1.58)	3.95 (1.10)	0.059	
Care (mean (SD))	2.27 (1.39)	2.50 (1.28)	0.61	
Lifting (mean (SD))	3.13 (1.36)	3.40 (0.99)	0.506	
Walking (mean (SD))	1.93 (1.44)	2.80 (1.40)	0.082	
Sitting (mean (SD))	1.80 (1.26)	2.90 (1.21)	0.013	
Standing (mean (SD))	2.53 (1.55)	3.35 (1.39)	0.111	
Sleeping (mean (SD))	2.67 (1.05)	3.00 (0.97)	0.339	
Social (mean (SD))	2.67 (1.54)	3.20 (1.11)	0.241	
Traveling (mean (SD))	2.00 (1.07)	2.95 (1.19)	0.02	
Housework (mean (SD))	2.47 (1.64)	3.00 (1.17)	0.269	
Score (mean (SD))	49.07 (22.68)	62.10 (17.79)	0.065	

Tertiary outcomes

Complications

No clinically significant complications were observed in this patient cohort. In 2/13 patients, we observed clinically silent cage subsidence, of which one patient had hardware connection loosening. This patient was followed until solid fusion was achieved. No notable complications were observed in this patient cohort.

## Discussion

In this study, we evaluated the surgical efficacy of MIS-DTIF procedure in thoracic spine degenerative disc disease (TS-DDD) patients whose clinical diagnosis included symptomatic thoracic herniated nucleus pulposus (TS-HNP) or thoracic discogenic stenosis. With the global advancement and evolution of medical technology, surgeons are becoming more open to the idea of adopting novel surgical techniques to improve the outcomes of surgically managed patients with spine problems. Over the past several decades, traditional open surgical approaches to thoracic spine herniation and stenosis patients have consisted of open-lateral or open-posterior approaches, which are highly invasive. These techniques include the removal of important structures in the spinal column, including rib bone (costectomy), facet bone (facetectomy), or partial resection of the rib head [4]. Even in the hands of experienced surgeons, invasive open procedures risk higher rates of perioperative complications for the patient, which include neurologic injuries in addition to pulmonary complications such as hemothorax and lung contusions. Due to the necessity for risk reduction in surgical thoracic spine patients, a minimally invasive spine procedure - MIS-DTIF - was developed by the first author [14]. In this technique, there is no open exposure or direct visualization of the posterior or lateral spine structures, which is complemented by MIS pedicle screw fixation that minimizes risk of lung injury encountered in traditional open approaches [4,5].

In this study, our team retrospectively reviewed perioperative measures of function, pain, and complication incidence in patients who underwent the MIS-DTIF surgical procedure. Demographically, the average age of patients in this cohort was 49.2 years, with five female patients and eight male patients. These findings imply that the female-to-male ratio of symptomatic TS-DDD or TS-HNP in the United States is potentially [1:1.6] and often occurs in middle-aged adults, though further data would be needed to verify these values. The average BMI of our MIS-DTIF patient cohort (30.46 kg/m2) falls within the Obesity Class I category and is consistent with the general obesity prevalence among U.S. adults aged 40-59 years, as reported by the National Health Statistics Report [20,21].

A study on traditional approaches to thoracic interbody fusion for disc herniation followed 12 patients after surgical treatment for 28 months and revealed an average hospital length of stay of five days [22]. When compared to our cohort of 13 patients who received treatment via MIS-DTIF, the average hospital stay was 1.1 days (Table 1). Further, our study revealed that the MIS-DTIF procedure is associated with substantially decreased surgery time compared to prior reported fusion surgery times [21]. This finding demonstrates strong evidence of the clinical and economic benefits the MIS-DTIF technique offers for patients who require thoracic spine surgery, as well as for healthcare providers.

Due to the anatomical differences between the upper and lower thoracic segments, the majority of thoracic disc herniation is seen below the T8 level - with greater than 75% of TS-DDD or TS-HNP occurring at T11 and/or T12 levels. This prevalence likely is related to the upper thoracic spine being less mobile due to the rib cage and other protective anatomical structures [4,5,23]. These statistics hold true in our studied cohort, with approximately 62% of our patients having TS-DDD or TS-HNP at the T8 vertebra or the levels below (Table 1).

We did not observe any clinically concerning complication events in this patient cohort. Analysis of the postoperative course for this cohort revealed three patients who required a chest tube postoperatively, as well as two patients who were noted to have either clinically silent hardware loosening and/or cage subsidence on postoperative imaging studies during follow-up clinic visits. It is worth noting that these patients remained asymptomatic and that none of them required reoperation for solid fusion to be achieved. Additionally, no patients in the study required reoperation throughout the entire study period.

The development and implementation of the MIS-DTIF technique were spurred by the need for an alternative to traditional thoracic spine surgery options for the management of TS-DDD or TS-HNP. Traditional procedures often expose patients to a higher risk of direct mechanical trauma, lung contusion, and other pulmonary complications that are often attributed to the inadvertent violation of the elements within the thoracic cavity while accessing the spine [24]. This is supported by a 2016 systematic review that evaluated pulmonary complication incidence in thoracic spinal surgery. They reported incidence findings of 0%-50% for pneumothorax and 0%-77% for both hemothorax and pulmonary effusion, respectively [24]. It is imperative to highlight that the justification for performing a minimally invasive spine surgery includes, but is not limited to, tissue preservation or reduced tissue damage. This results in a quantifiable clinical benefit (as reported with blood loss volume, decreased surgical morbidity and hospital stay, and early resumption of regular activities) as well as clinical and cost-effectiveness [25].

While reviewing the literature, we noted a study by Deviren V. et al., which introduced a different novel minimally invasive thoracic spine surgery. This approach utilized an anterolateral transthoracic transpleural approach for managing TS-HNP patients and reported a postoperative complication incidence of 16.67% (n=2/12). Notably, one of their two patients had pleural effusion, and the other patient had intercostal neuralgia in their studied cohort. In comparison, we can appreciate the benefits of tissue preservation with the MIS-DTIF approach, which confers clinical, safety, and economic benefits for the management of symptomatic thoracic disc herniation or stenosis patients. This is important in a patient population that is potentially vulnerable to 100% thoracic visceral violation with the potential to result in reoperations, increased morbidity, and increased hospital costs [22].

Apart from clinical efficacy, the MIS-DTIF approach has a less-steep learning curve than other thoracic spine surgery modalities, and can conveniently allow tricalcium phosphate (TCP) or other biological packing to be introduced into the prepared disc spaces during the fusion procedure. This can be especially beneficial, as narrow disc entrances within the thoracic spine can make operation in this region technically challenging and more traumatic [26-28]. It is important to note that only three of the patients in our cohort required a chest tube (typically removed after one day), which is much less than the rate and duration of chest tube placement after a thoracotomy is used to access the spine [29]. Further, traditional open thoracic fusion surgery usually requires interdisciplinary surgical assistance, such as a cardiothoracic or vascular surgeon, to be present at the time of surgery [29]. Due to the decreased intraoperative risk associated with the MIS-DTIF technique - and an approach that aims to avoid major thoracic vascular structures - this requirement is not needed and was not utilized in our study. Despite utilizing a smaller surgical team than traditional open thoracic fusion surgery, at a minimum average follow-up period of 12.1 months, we report 0% incidence of clinically significant complication or reoperation rates in our cohort (Tables 1, 2). Comparatively, this is an impressive statistic since the frequency with which reoperation is needed in traditional, invasive thoracic spine procedures is well acknowledged [28]. Larger multi-institutional studies are warranted to validate and ascertain these findings.

Strengths and limitations

The key strength of this study is that it is the first report for this surgical modality on 13 patients - about 69.2% more patients than the initial proof of concept study - which followed four individuals treated with MIS-DTIF for the management of TS-DDD or TS-HNP. This adds to the relevant body of spine literature by highlighting the efficacy and safety of the MIS-DTIF approach and encourages spine surgeons to consider utilizing the MIS-DTIF approach as a standard surgical treatment - or viable option - for the treatment of TS-DDD or TS-HNP patients. Apart from increased procedural safety, clear statistically significant improvements in VAS pain scores following the operation emphasize surgical efficacy in pain reduction. This reduced pain also translated to improved overall ODI scores - particularly in “sleep” and “return to work” domains - highlighting the importance of pain reduction on quality of life. 

Our study is a retrospective review of cases and, therefore, not without limitations. Notably, three of our patients had incomplete postoperative ODI patient-reported outcomes due to failed attempts in contacting patients who had received MIS-DTIF surgery and a lack of patient follow-up in the clinic. To address this gap, calculated means and standard deviations in our statistical analysis were focused on representing the fraction of patients with complete perioperative patient-reported outcomes. Finally, a follow-up study with an increased population of patients would increase power analysis and delineate more clear clinical outcomes of the MIS-DTIF procedure. We hope to accomplish this in the near future - since the execution of a follow-up study would be contingent on the number of MIS-DTIF cases performed.

## Conclusions

Findings from this study demonstrate that the MIS-DTIF surgical approach is not only efficacious for the management of patients with thoracic spine degenerative disc disease but also has a good safety profile. This was achieved by avoiding thoracic viscera violation, which translated to no occurrences of hemothorax or pleural effusions in our presented cohort. Additionally, our MIS-DTIF patient cohort demonstrated significant improvement in back pain intensity and disability outcomes as measured by VAS and ODI scores, respectively. With growing interest in value-based clinical models in healthcare, we anticipate that the adoption of the MIS-DTIF surgical technique will benefit spine patients and improve their pain and functional outcomes both short- and long-term following surgical treatment. The MIS-DTIF approach is therefore warranted as a standard, clinically safe surgical procedure for effectively managing spine patients diagnosed with TS-DDD or TS-HNP.
